# Peer Mentoring to Foster Resilient Age-Friendly Rural Communities in Maine

**DOI:** 10.1093/geroni/igab046.1621

**Published:** 2021-12-17

**Authors:** Patricia Oh, Jennifer Crittenden, Laura Lee

**Affiliations:** 1 UMaine Center on Aging, Bangor, Maine, United States; 2 University of Maine, University of Maine, Maine, United States; 3 Maine Community Foundation, Ellsworth, Maine, United States

## Abstract

Maine has a growing number of age-friendly community initiatives (AFCIs); 116 communities are actively working to adapt the social, service, and built environments for aging and 71 have formally joined the AARP Network of Age-Friendly States and Communities. During COVID, rural municipalities were faced with dynamic changes that limited older resident’s access to services and social engagement. To overcome these limitations, it is critical for emergent AFCIs to have tools and strategies to maintain and further enhance healthy environments and resilient communities. This study uses group interviews with 6 leaders of established AFCIs and 6 leaders of emergent AFCIs to explore how the Lifelong Fellows Program, a peer mentoring model that matches experienced leaders with newly formed initiatives, was able to spur development of new strategies to build community resilience. Prominent themes were (1) engaging new local and regional partners; (2) intergenerational volunteerism; (3) fun and flexibility; and (4) relationship-building.

